# Pet Food Factory Isolates of* Salmonella* Serotypes Do Not Demonstrate Enhanced Biofilm Formation Compared to Serotype-Matched Clinical and Veterinary Isolates

**DOI:** 10.1155/2019/8569459

**Published:** 2019-01-29

**Authors:** Amreen Bashir, Ansar Azeem, Yvonne Stedman, Anthony C. Hilton

**Affiliations:** ^1^School of Life & Health Sciences, Aston University, Birmingham, B4 7ET, UK; ^2^Mars, Incorporated, McLean, VA, USA

## Abstract

Environmentally persistent* Salmonella* in the pet food factory environment has been described, with biofilm formation suggested as a candidate mechanism contributing to their persistence. In this study the ability of a panel of* Salmonella* isolates from factory, clinical, and veterinary sources was investigated for their ability to form biofilms at 24 and 48 hours. The effect of nutrient availability and incubation time on biofilm formation was investigated using full strength and diluted 1/20 TSB media at 37°C, 25°C, 15°C, and 10°C. Results highlighted that all the* Salmonella* isolates were able to form biofilms in both nutrient conditions and this was highly correlated with temperature. At 25°C, biofilm formation was enhanced in diluted 1/20 TSB and increased incubation time (48h) (p= <0.001). However, this was not observed at 10°C, 15°C, or 37°C. None of the factory isolates demonstrated enhanced biofilm formation in comparison to serotype-matched isolates from veterinary and clinical sources.* Salmonella enterica* Senftenberg 775W was the strongest biofilm former at 15°C, 25°C, and 37°C in all the conditions tested (p=<0.05). Biofilm formation is an important mechanism of environmental persistence in the food manufacturing environment; however, there is no evidence of an enhanced biofilm-producing phenotype in factory persistent strains.

## 1. Introduction

An important factor enabling environmental survival of microorganisms, especially in nutrient depleted conditions, is their ability to form biofilms [1, 2]. A biofilm is classified as a population of microbial cells that is associated with a surface and enclosed in a matrix of primarily polysaccharide material [3]. The cells in a biofilm produce proteinaceous substances which allow protection from environmental stresses.

Reports have highlighted the presence of problematic, persistent microorganisms such as* Salmonella, L. monocytogenes,* and* E. coli *in the microflora of the food manufacturing environment and suggested that persistence of the pathogens may be contributed to by multiple mechanisms [4, 5].

The protective nature of the biofilm makes it a candidate mechanism to explain the environmental persistence observed in some food factory isolates of* Salmonella *[6]. Biofilm formation can occur where microorganisms and surfaces are in contact [7], and in the food industry this is clearly problematic in relation to their control. Biofilms are difficult to control in areas of the factory environment where effective cleaning is compromised [8]. In addition to persistence, detached cells from the biofilm can lead to disseminated contamination of the wider production environment and food products.* Salmonella* spp. have been reported to form biofilms on a range of surfaces found in the food manufacturing environment including plastic waste water pipes [7, 9], glass [10], concrete floors [11], and stainless steel [7, 12, 13]. The development of biofilms on surfaces has been suggested to be one of the principal mechanisms for the survival and persistence of* Salmonella* in food manufacturing environments, and some strains have been reported to survive on the surface of equipment for many years [7, 14].

Environmental persistence of* Salmonella *has been associated with their ability to form biofilms [7, 12]; however, it is currently unknown if pet food factory isolates demonstrate an enhanced capability compared to* Salmonella* adapted to other environments. The aim of this study therefore was to establish the biofilm forming capacity of a panel of* Salmonella* isolates at different temperatures and duration of incubation in both nutrient-rich and nutrient-deprived media. Having a better understanding of the role of biofilms as a potential mechanism of persistence of food factory isolates will provide valuable data necessary to control their persistence in food manufacturing environments.

## 2. Material and Methods

### 2.1. Bacterial Strains

A panel of ten* Salmonella* was created comprising isolates known to be persistent in the pet food factory environment, veterinary, and well-characterised reference strains (Table 1).* Listeria monocytogenes *(NCTC11994) was included as a strong biofilm-forming positive control. The factory isolates originated from environmental swabs collected at two pet food manufacturing sites producing dry complete pet food and wet food in tins and pouches. Environmental swabs were collected daily at 20-40 sample points based on factory size, number of systems, and subprocesses. Persistent strains were defined as those isolated repeatedly from designated sample points within the factory on more than eight independent occasions. All swab locations were mapped and documented as part of the factory master sanitation program.

Swabbing was conducted using sterile, cellulose sponge swabs premoistened with 10ml sodium thiosulphate buffer and containing neutralizers of Tween 80 and Lecithin (TSC Ltd., Lancashire, UK). Environmental swabs were collected daily at 20-40 sample points based on factory size, number of systems, and subprocesses. All swab locations were mapped and documented as part of the factory master sanitation program. Pet food factory isolates included* S*. Senftenberg,* S*. Livingstone,* S*. Kedougou,* S*. Montevideo, and* S*. Schwarzengrund (USA). As far as possible isolates from the different environments were serotype matched. Veterinary strains from canine isolates were obtained from the Veterinary Laboratory Agency, Surrey, UK (VLA), and included* S.* Senftenberg (VLA) and* S*. Schwarzengrund (VLA).* S*. Schwarzengrund, USA, caused an outbreak associated with pet food in 2009 and the* S. *Schwarzengrund (FSL S5-458) is the American clinical isolate which was isolated from patients during the outbreak. The heat resistant strain* S. *Senftenberg 775W is well-documented and unlike other strains it is a nonhydrogen sulphide producer. Globally,* S*. Senftenberg 775W (ATCC 43845) is not a major cause of salmonellosis but outbreaks are commonly associated with contaminated poultry and plant derivative food.* S*. Typhimurium SL1344 was included in the panel as it has been typed and literature shows that the serotypes Typhimurium and Enteritidis are the leading cause of* Salmonella* disease. All were stored on Microbank beads (Fisher Scientific, UK) and maintained at -80°C until required.

### 2.2. Pet Food Factory Environmental Monitoring

#### 2.2.1. Measurement of Relative Humidity and Ambient Air Temperature

The relative humidity (RH) and ambient temperature of a factory producing heat extruded product subject to ambient cooling were monitored every 10 minutes for two months (May–July) using a Hygropalm-HP21 data logger (Rotronic, West Sussex, UK). The manufacturing cycle was four-day production followed by three-day shutdown. These environmental data were used to inform the incubation temperature of the biofilm production study.

#### 2.2.2. Biofilm Assay

The* Salmonella* biofilms were grown in sterile polystyrene 96-well flat microtitre plates (Fisher Scientific, UK), using a method as described by Stepanovic et al. [13]. Full strength Tryptone Soya Broth (TSB; Oxoid, Basingstoke, UK) and 1/20 TSB were prepared according to manufacturer's instructions and sterilised by autoclaving at 121°C for 15 minutes. Prepared media were stored at 4°C until required. A 230*μ*l volume of neat TSB and 1/20 TSB were added to the wells in the microtitre plate. To prepare the standardised inoculum, a Microbank bead carrying each strain was recovered from frozen storage and added to a Universal tube containing 20ml of fresh TSB culture media. This was incubated with shaking for 18–24hrs at 37°C. Following incubation, a stock inoculum was prepared by taking 5 ml of the TSB media and adding to 5 ml of fresh TSB media in a sterile universal tube. The stock inoculum was mixed by vortexing for 60 seconds. A 1ml volume of the stock inoculum was transferred into a disposable cuvette (Sigma-Aldrich, UK) and the optical density (OD) at 600nm was noted. The stock inoculum was diluted as required by the addition of fresh TSB media to generate a standardised inoculum concentration of 10^6^ cfu/ml as established by previous OD calibration studies (data not shown). A 20*μ*l volume of standardised inoculum was added to the 230*μ*l volume of neat TSB and 1/20 TSB in the microtitre plate and incubated statically at 37°C, 25°C, 15°C, and 10°C for 24hrs or 48hrs as required. For the 48hr plates the culture media were changed for fresh TSB or 1/20 TSB as appropriate, at 24hrs; the spent culture media was removed with a pipette and 250*μ*l fresh media added and the plates returned to incubation for a further 24 hours before being assayed for biofilm production.

The biofilm assay was undertaken on 24hr and 48hr incubated plates. The spent culture media were removed with a pipette and each well was washed twice by gentle irrigation with 300*μ*l of sterile distilled water (SDW). A 250*μ*l volume of 100% methanol (Fisher Scientific, UK) was added to each well to fix the bacteria and incubated for 15 minutes before the methanol was removed with a pipette. The plates were then air dried by incubation at ambient temperature to evaporate the remaining methanol. A 250*μ*l volume of crystal violet (CV) dye (Sigma-Aldrich, UK) was added to each well and biofilm biomass determined by crystal violet assay according to Stepanovic et al., 2014). The optical density at 570nm of the liberated CV was measured using a BIOTEK Elx808 Absorbance micro plate reader (Biotek, UK) and the OD data exported for statistical analysis using ANOVA in STATISTICA version 10 (USA). Each experiment was repeated four times.

### 2.3. Statistical Analysis

An ANOVA was conducted to investigate the differences across the four temperatures. Further data mining included extracting the serotype-matched data for the clinical, factory, and veterinary isolates of* S*. Schwarzengrund and* S*. Senftenberg and conducting an ANOVA to explore if any environment driven effects were present.

## 3. Results

A summary description of the relative humidity and ambient temperature of the pet food factory environment monitored over a 60-day period is presented in Table 2. The average recorded temperature was 21.5°C with 54.5% RH. The maximum temperature reached was 29.2°C with 79.3% RH and the minimum temperature was 15.6°C with 30.5% RH. The average temperature recorded in the packaging room was 18.6°C with 56.2% RH. The maximum in this zone reached 26.2°C with 72.8% RH and the minimum temperature fell to 14.7°C with 34.6% RH during factory shutdown.

**Figure 1:**
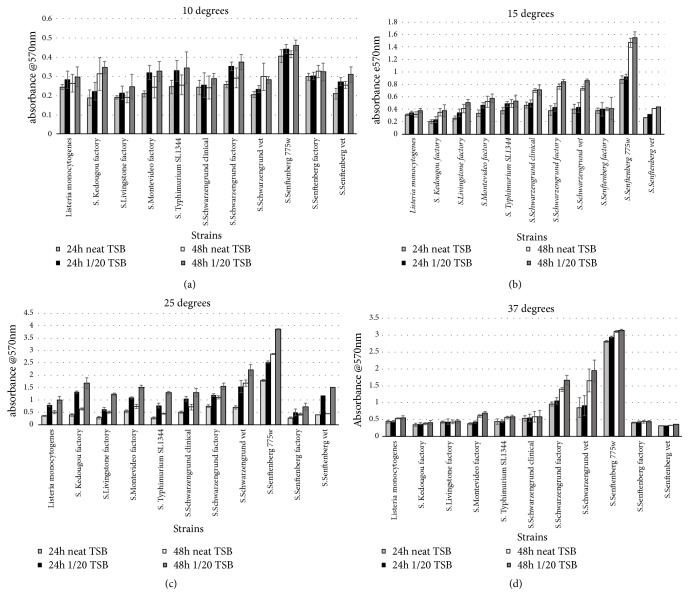
*Biofilms*. Mean 24h and 48h biofilm density measurements for each strain at 37°C, 25°C, 15°C, and 10°C in neat TSB and 1/20 TSB media. Error bars represent standard deviation of the mean.

The mean 24h and 48h biofilm density measurements for each strain at the various temperature and media concentrations investigated are shown in Figures 1(a)–1(d). All the strains in the panel could form biofilm with* S. *Senftenberg 775W being the strongest biofilm producer at 37°C, 25°C, and 15°C. At all temperatures investigated; no association was observed (p=>0.05) between the environmental source of the isolate and the biofilm density. As a general trend, stronger biofilms were produced as the temperature of incubation increased from 10°C to 37°C.

At 25°C across the panel of isolates there was a significantly more established biofilm at 48h compared to 24h and at 1/20 TSB compared to neat TSB media (P=<0.01). This effect of time and nutrient depletion on biofilm production was not observed at the other temperatures investigated. A chi square test revealed that at 10°C and 37°C there were no statistically significance differences between the ability of isolates to form biofilms in neat or 1/20 TSB media and increased incubation time (p=>0.05). Analysis at 15°C revealed that all isolates except* S. *Senftenberg factory produced statistically stronger biofilms with 48h incubation (p=<0.01). Furthermore all of the isolates produced statistically stronger biofilms in diluted media compared to full strength at 24 hours (P=<0.01) with the exception of* S. *Senftenberg factory and* S*. Typhimurium SL1344.

Comparison of the serotype-matched clinical, factory, and veterinary isolates of* S*. Schwarzengrund and* S*. Senftenberg revealed that S. Senftenberg 775W was the strongest biofilm producer across 15°C, 25°C, and 37°C (p=<0.05). Both of the factory isolates did not demonstrate an enhanced capacity to produce biofilms in comparison to serotype-matched isolates in the neat and 1/20TSB media conditions. No other effect of serotype or strain origin was observed across the six isolates tested (p=0.067).

## 4. Discussion

*Salmonella *is able to persist in the food manufacturing environment for years [15]. Biofilms are particularly problematic as they represent a persistent focus of* Salmonella* which is difficult to control and can be a source of disseminated, postprocess contamination. Previous studies have highlighted the role of* Salmonella* contamination of factory surfaces and the production environment [16–18]. However, there is limited knowledge on the underlying mechanisms by which environmentally adapted factory isolates of* Salmonella *may demonstrate enhanced survival in comparison to those from other environments. This study investigated a panel of ten defined isolates of* Salmonella* originating from the pet food factory, veterinary, and clinical environment by comparing their ability to form biofilms attached to surfaces. The effect of temperature, incubation time, and media concentration on biofilm formation was investigated.

Joseph et al. [11] investigated the ability of poultry isolates of* Salmonella* to form biofilms on stainless steel, plastic, and cement and found that the highest density of biofilm formed on plastic, followed by cement and finally stainless steel. Other studies also indicated that* Salmonella* and* L. monocytogenes* adhere in higher numbers to hydrophobic materials such as plastic [3, 19]. Considering adhesion is the primary step in biofilm formation, it could explain why all the isolates investigated in this study are able to form good biofilm on plastic surfaces [13].

The incubation temperatures used in the currently described study were selected to model those typical of a large UK-based pet food manufacturing environment, and, with exception of the* S. *Schwarzengrund, the same environmental conditions from which the factory strains used in this study were isolated. These realistic environmental conditions represented temperature fluctuations during periods of production and shut-down. During the factory shut-down period temperatures fell to 15°C and during production the heat processing through cooling and packaging zones of the factory showed temperatures ranging from 15°C to 29°C, with the highest temperature in the packaging zone being 26°C. Other studies, modelling and predicting the biofilm capabilities of* Salmonella* and investigations into the survival of* Salmonella*, have also been conducted at similar temperatures to represent conditions present in dry food manufacturing plants [17, 20, 21].

All of the* Salmonella* isolates investigated could form biofilms, independent of the environment from which they were isolated, and the strength of biofilm formation was not directly linked to serotype (Figures 1(a)–1(d)). One notable exception to this was* S*. Senftenberg 775W which, with the exception of 10°C (Figure 1(a)), under all other conditions investigated produced significantly stronger biofilms compared to the other* Salmonella* isolates (p=<0.05).* S. *Senftenberg 775W is a known heat resistant strain and on this basis is frequently used as a challenge organism in the food industry [22, 23]. The underlying mechanism of the enhanced heat resistance demonstrated by* S.* Senftenberg 775W is poorly understood; however, on the basis of observation made here, it is likely at least some of its resistance properties may be attributed to its ability to form stronger biofilms compared to other more sensitive salmonellae.

Setting* S.* Senftenberg 775W aside as a clear outlier, none of the remaining* Salmonella* isolates, from any source, demonstrated an enhanced ability to generate biofilm under any of the conditions investigated. Whilst the survival and persistence of* Salmonella* in the food factory environment have been the subject of previous investigation, their survival in comparison to serotype-matched clinical and veterinary isolates has not been reported previously [14, 17, 24].

In the current study, resident pet food factory strains did not produce significantly stronger biofilms in comparison to clinical and veterinary isolates of the same serotype. Although all the isolates could form biofilm, and formation of biofilm is likely to be advantageous in both the clinical and veterinary environment, it does suggest that factory isolates are no more capable of forming biofilm than their serotype-matched counterparts. Vestby et al. [21] compared biofilm production of “persistent” and “nonpersistent” strains of* S*. Agona and* S*. Montevideo, reporting that the “persistent” strains were stronger biofilm producers than the “nonpersistent” strains; however, the matched isolates studied were also from the same factory environment, suggesting that biofilm formation was more linked to serotype than the environment from which it was isolated.

Interestingly, biofilm formation was the highest at 25°C, followed by 37°C and decreased, respectively, at 15°C and the lowest level of biofilm formation was seen at 10°C. However, the ability to produce biofilms is not exclusively linked to growth as formation of biofilm at 37°C was not as strong as at 25°C, and it would be anticipated that the growth rate of* Salmonella *would be higher at 37°C than at 25°C. At lower temperatures the strains were unable to form as strong biofilms, presumably as cells struggled to grow; if cells were unable to grow they could not attach to a surface and grow in sufficient number to produce a substantial extracellular matrix. Similarly, a study by Tammakritsada and Todhanakasem [25] investigated the ability of* Salmonella *to form biofilms on polystyrene tubes and also showed the same pattern with the density of biofilm formation decreasing with temperature from 25°C to 15°C and finally to 10°C. In the current study, only at 25°C was an enhanced biofilm observed after 48 hours in comparison to that formed at 24 hours, which supports the observations of others [13]. Vestby et al. [14] reported that* Salmonella* biofilm formation was favoured at 20°C and could be linked to its persistence in fish meal and feed production environments. The observation that* Salmonella* produced enhanced biofilm at 25°C compared to the other temperatures investigated in this study identifies a risk factor for environmental persistence. As* Salmonella* is able to form biofilms on surfaces and survive for months at 25°C, which is close to factory ambient temperatures, this poses an enhanced risk of cross-contamination within the manufacturing environment.

Bacteria in food processing environments are likely to be exposed to differing levels of available nutrients depending on their location in the factory plant [8]. In laboratory studies it has been observed that the concentration of the culture media is an important variable in influencing biofilm formation for both* Salmonella* and* L. monocytogenes *[13]. Furthermore time is also an important parameter in biofilm development; the longer the bacterial cells take to form biofilms, normally the more comprehensive and dense the biofilm. Stepanovic et al. [13] investigated biofilm formation in four media types at 35°C over 24 hours; brain heart infusion (BHI), trypticase soy broth (TSB), meat broth (MB), and 1/20 diluted trypticase soya broth. They reported that* Salmonella* formed better biofilms in low nutrient diluted TSB media, used to mimic factory conditions, in comparison to full strength TSB. In a similar study by Paz-Mendez et al. [24] in which they investigated the effect of food residues on biofilm formation it was found that 1/20 diluted TSB media enhanced the development of biofilm in all* Salmonella* isolates investigated

In the current study, biofilm formation in 1/20 TSB media was compared to that in full strength TSB media at four temperatures and the effect of increased incubation to 48 hours following a media change was also investigated. With the exception of 25°C, where across the panel of isolates there was a significantly more established biofilm at 1/20 TSB compared to full strength (P=<0.05), at the other temperatures although similar trend was revealed whereby 1/20 TSB media promoted biofilm development; this did not achieve significance (p=>0.05). The general observation of enhanced biofilm development in 1/20 TSB media over a range of incubation temperatures is consistent with that of Paz-Mendez et al. [24]; however, low nutrient availability may not be a significantly independent factor in promoting biofilm development but influenced by temperature and serotype.

## 5. Conclusion

Biofilm formation is an accepted mechanism facilitating the persistence of* Salmonella* in the environment and enhanced resistance to disinfection. This study highlighted that all the isolates in the challenge panel were able to form biofilms in both nutrient-rich and nutrient-limited environments with higher levels of biofilm production occurring at 25°C and 37°C. At 37°C, 15°C, and 10°C an extended duration of incubation had no general effect on the ability of strains to form more established biofilms; however, at 25°C biofilm formation was significantly enhanced at 48 hours. Under all conditions investigated, although all were able to form biofilm, none of the factory isolates showed an enhanced capability to form biofilms in comparison to serotype-matched isolates from veterinary and clinical sources. Biofilm formation continues to represent an important mechanism of environmental persistence of* Salmonella* in the food manufacturing environment; however, there appears to be no evidence of an enhanced biofilm-producing phenotype in factory persistent strains compared to serotype-matched isolates from nonmanufacturing environments.

## Figures and Tables

**Table 1:** * Challenge panel of isolates. * Panel of isolates selected for a majority of the investigations detailing the origin of the isolate. Serotype-matched clinical and veterinary isolates were sourced for the pet food factory isolates of *S*. Senftenberg and *S*. Schwarzengrund to balance serotypes.

**Strain**	**Source**
***S*. Senftenberg 775W**	ATCC 43845
***S*. Senftenberg **	Pet food factory UK
***S. *Senftenberg**	VLA
***S*. Schwarzengrund**	FSL S5-458 American clinical
***S*. Schwarzengrund**	Pet food factory USA
***S*. Schwarzengrund**	VLA
***S*. Typhimurium SL1344**	NCTC 13347
***S*. Livingstone**	Pet food factory UK
***S*. Kedougou**	Pet food factory UK
***S*. Montevideo**	Pet food factory UK
***L. monocytogenes***	NCTC 11994

**Table 2:** *Environmental sampling of temperature and relative humidity in the preparation and packaging zones of the factory*. Temperature and relative humidity profiles of the preparation and packaging room over a 60-day period. Monitoring was undertaken at 10-minute intervals.

	**Preparation room**		**Packaging room**	
	Temp °C	%RH	Temp °C	%RH
AVERAGE	21.5	54.5	18.6	56.2
STD DEVIATION	2.5	7.5	2.6	9.5
MAXIMUM	29.2	79.3	26.2	72.8
MINIMUM	15.6	30.5	14.7	34.6
MODE	21.2	54.9	16.6	63.1

## Data Availability

The data used to support the findings of this study are available from the corresponding author upon request.
